# Citrus Peel Extract Ameliorates High-Fat Diet-Induced NAFLD via Activation of AMPK Signaling

**DOI:** 10.3390/nu12030673

**Published:** 2020-03-01

**Authors:** Geum-Hwa Lee, Cheng Peng, Seon-Ah Park, The-Hiep Hoang, Hwa-Young Lee, Junghyun Kim, Seong-Il Kang, Chi-Heon Lee, Joo-Sang Lee, Han-Jung Chae

**Affiliations:** 1 Non-Clinical Evaluation Center, Biomedical Research Institute, Chonbuk National University Hospital, Jeonju, Chonbuk 54907, Korea; heloin@jbnu.ac.kr (G.-H.L.); pc772052@gmail.com (C.P.); topaz6183@jbnu.ac.kr (S.-A.P.); drhiep.ydhue@gmail.com (T.-H.H.); youngat84@gmail.com (H.-Y.L.); 2 Research Institute of Clinical Medicine of Chonbuk National University-Biomedical Research Institute of Chonbuk National University Hospital, Jeonju, Chonbuk 54907, Korea; 3 Department of Pharmacology, Chonbuk National University Medical School, Jeonju, Jeonbuk 54896, Korea; 4 Department of Oral Pathology, School of Dentistry, Chonbuk National University, Jeonju, Chonbuk 54896, Korea; dvmhyun@jbnu.ac.kr; 5 Jeju Institute of Korean Medicine, Jeju 63309, Korea; sikang@jikom.or.kr (S.-I.K.); leechiheon@jikom.or.kr (C.-H.L.); jejuherb@jikom.or.kr (J.-S.L.)

**Keywords:** citrus peel, NAFLD, high-fat diet, mTOR-ER stress, AMPK

## Abstract

Non-alcoholic fatty liver disease (NAFLD) is prevalent in the elderly population, and has symptoms ranging from liver steatosis to advanced fibrosis. Citrus peel extracts (CPEs) contain compounds that potentially improve dyslipidemia; however, the mechanism of action and effects on hepatic steatosis regulation remains unclear. Current study was aimed to investigate the protective effect of CPEs extracted through hot-air drying (CPEW) and freeze-drying (CPEF) and the underlying mechanism in a rat model of high-fat diet-induced NAFLD. The high-fat diet (HFD)-fed rats showed significant increase in total cholesterol, alanine aminotransferase (ALT), triglycerides, aspartate aminotransferase (AST), and lipid peroxidation compared to the normal chow-diet (NCD) group rats; but CPEW and CPEF limited this effect. CPEW and CPEF supplementation reduced both hepatocyte steatosis and fat accumulation involving the regulatory effect of mTORC1. Collectively, CPEW and CPEF protected deterioration of liver steatosis with AMPK activation and regulating ROS accumulation associated with interstitial disorders, which are also associated with endoplasmic reticulum (ER) redox. Thus, the application of CPEW and CPEF may lead to the development of novel therapeutic or preventive agents against NAFLD.

## 1. Introduction

Since ancient times, plants are widely used for their medicinal values and are valuable sources of new drugs that are developed to treat various diseases. The previous researches have elucidated that several compounds of *citrus* plants possess a wide range of pharmacological effects, such as anti-inflammation, antioxidation, anticancer, and neuroprotection [1,2]. Specifically, the dried peel of citrus, a functional health food ingredient, is used as a traditional herbal ingredient to treat various digestive dysfunctions, involving dyspepsia, abdominal distension, and vomiting in East Asia [3]. Further, few in vivo studies on citrus extracts have indicated their ameliorative effects in disturbances of gastrointestinal microbiota and obesity conditions. To date, the citrus peel is not functionally identified as antidyslipidemic, as well as for non-alcoholic fatty liver disease (NAFLD). Many epidemiological studies have shown that NAFLD is a complex metabolic disorder that plays a critical role in the progression of liver lesions associated with excess intake of nutrition and energy, resulting in insulin resistance, dyslipidemia, and chronic inflammation. These clinical conditions are characterized by excessive fat deposition in the liver, presenting a wide range of histological conditions such as simple steatosis to nonalcoholic steatohepatitis (NASH) [4]. Emerging studies reveal that hepatic dyslipidemia is one of the key contributors to increased fat mass and triglyceride accumulation, while controlled lipid metabolism protects against the development of NAFLD [5]. In NAFLD, hepatic fat accumulation is mediated by de novo lipidogenesis (DNL) and increased uptake of fatty oxidation [6]. For further development of NAFLD, excessive influx of fatty acid to liver is believed to be pathogenic cause and this influx is mediated by fatty acid transport proteins (FATP), caveolins and CD36 cluster of located in the hepatocyte plasma membrane [7]. However, mechanism which regulates lipid homeostasis and NAFLD are complex and interconnected. Among controlling mechanisms against NAFLD, AMP-activated protein kinase (AMPK) play a crucial role in preventing hepatic lipid accumulation in high-fat diet (HFD)-fed mice and excess nutrient-treated HepG2 [8,9]. In addition, AMPK activation inhibits the mTORC1 activation and ER stress response [8]. Recent studies have also indicated that alteration of AMPK/mTOR pathway activity is involved in steatosis, insulin resistance, and modulation of lipid metabolism in HFD induced mice by stimulation of autophagy [10,11]. Thus, the inhibition of hepatic dyslipidemia via AMPK activation is considered as a viable therapeutic strategy in prevention of occurrence and development of NAFLD [12]. In view of the potential therapeutic value of CPEs, this investigation aimed to assess the antidyslipidemic efficacy of CPE by determination of lipid metabolism dysfunction in HFD-administered rats, to open the possibility of CPE as a potential antidyslipidemic functional food/plant.

## 2. Materials and Methods

### 2.1. AML 12 Cell Culture

Mouse hepatocyte cells (AML 12) cells were maintained in DMEM/F-12 medium (Gibco, Grand Island, NY, USA) supplemented with 10% fetal bovine serum, 40 ng/mL dexamethasone (Sigma-Aldrich, St. Louis, MO, USA) and 1xITS (insulin/transferrin/selenium, Gibco). Cells were cultured at 37 °C in 5% CO_2_ incubator. Later, upon reaching 85–90% confluence in 6 cm dishes, they were incubated with 250 μM of palmitate with or without CPEW and CPEF extract (25, 50, and 100 μg/mL) for 24 h.

### 2.2. Citrus Peel Extract

Dried peels of organically cultivated *Citrus unshiu* was obtained from Jeju, Republic of Korea and crushed with a roll mill. Crushed peels (750 g) were added with 10 L of distilled water and extracted for 3 h at 100 °C. The CPEs were centrifuged and the supernatant was filtered using filter paper. Later, with a rotary evaporator, the filtrates were concentrated and then with a freeze dryer, the concentrates were lyophilized. The yield of the freeze-dried CPE extract (CPEF) was 34.13%. Similarly, to obtain air dried CPE, filtrate was concentrated in a rotary evaporator and then lyophilized with hot air-drying for 72 h at 50 °C. The yield of the dried CPE extract (CPEW) was 38.13%.

### 2.3. Quantification of the Main Components of Citrus Peel Extract Using High-Performance Liquid Chromatography with Diode-Array Detection (HPLC-DAD)

Agilent 1260 Infinity II HPLC system (Agilent Technologies, Wilmington, DE, USA) with a DAD detector was employed for analysis. 10 μL of the processed samples were injected into a HPLC system fitted with Atlantis^®^ T3 (4.6 × 250 mm, 5 μm). A mobile phase composed of 0.1% phosphoric acid in distilled water (Solvent A) and 0.1% phosphoric acid in acetonitrile (Solvet B) was used to separate the analytes and then pumped onto the column at a flow rate of 1 mL/min for 65 min. Injection volume was set at 10 μL and detection was carried out at 210 nm.

### 2.4. Ethics Statement

Animal welfare and experimental procedures were performed strictly in accordance with the Care and Use of Laboratory Animals (Jeonbuk National University IACUC) and obtained prior approvals for handling of animals (cuh-IACUC-2019-09).

### 2.5. Animal Grouping and Experimental Protocol

In this study, 56 male SD rats (eight-week-old) were purchased from Orient Science Co. (Seongnam, Korea). The rats were housed under standard living conditions where temperature is maintained at 22 ± 2 °C with a 12-h light/dark cycle and 55% to 60% relative humidity. Rats were acclimatized to laboratory conditions for a week before their use in the experiments. Standard diet was provided to normal chow diet (NCD) group, whereas high-fat diet (HFD) group were fed with calorie-rich diet consisting of 18% lipid, 1% cholesterol, vitamins (AIN 93G), 19% casein and 40% sucrose with similar mineral and fibers as NCD groups diet. This study comprised of 7 groups and each had eight experimental animals. Group 1, NCD-group: rats received double-distilled water (DDW); group 2, 60% HFD group: rats received DDW; group 3, 60% HFD plus CPEW-group: rats received DDW with CPEW (50 mg/kg); group 4, 60% HFD plus CPEW-group: rats received DDW with CPEW (100 mg/kg); group 5, 60% HFD plus CPEF-group: rats received DDW with CPEF (50 mg/kg); group 6, 60% HFD plus CPEF-group; rats received DDW with CPEF (100 mg/kg); group 7, 60% HFD plus metformin-group: rats received DDW with metformin (100 mg/kg).

### 2.6. Biochemical Analyses

Plasma levels of low-density lipoprotein (LDL) cholesterol (Biovision, Milpitas, CA, USA), triglycerides (TG), total cholesterol, alanine aminotransferase (ALT), aspartate aminotransferase (AST, Asan Pharmaceutical, Seoul, South Korea) were determined using commercially available kits. Hepatic tissue was homogenized and was extracted from a mixture of chloroform, methanol, and distilled water. Concentration of TG (Asan Pharmaceutical, Seoul, South Korea), levels of catalase (CAT), glutathione peroxidase (GPx) activity, and superoxide dismutase (SOD) were determined using a commercially available assay kit (Cayman Inc., Ann Arbor, MI, USA).

### 2.7. Western Blotting

Liver and cell lysates were separated by 4–12% sodium dodecyl sulfate polyacrylamide gel electrophoresis (SDS-PAGE) and transferred to an immunoblot polyvinylidene difluoride (PVDF) membrane using a semi-dry transfer system (Bio-Rad, Hercules, CA, USA). After blocking with 5% BSA or 5% skim milk, the blot was probed with primary antibodies against anti-p-p70s6 kinase, anti-p70s6 kinase, anti-p-4EBP-1, anti-4EBP-1,anti-p-mTOR, anti-mTOR, anti-p-eIF2α and anti-eIF2α, and anti-CHOP (Cell Signaling Technology, Inc., Danvers, MA, USA), Anti-p-PERK, anti-PERK, and β-actin (Santa Cruz Biotechnology, CA, USA).Westernblots were visualized using the enhanced chemiluminescence (ECL) system (Bio-Rad, Hercules, CA, USA).

### 2.8. Cellular Production of ROS Using Dihydroethidium

Intracellular ROS including O2^-^ was detected using an oxidative-sensitive fluorescent probe dye, dihydroethidium (DHE, Molecular Probes, Eugene, OR, USA). Tissue samples were washed with PBS and incubated with 20 μM DHE for 30 min at 37 °C. For quantification, tissue sections were scanned with a 20 × objective lens. Fluorescence intensity areas were quantified with DHE staining using a Zeiss LSM 510 META confocal microscope (Carl Zeiss, Jena, Germany), subtracting the background fluorescence.

### 2.9. Analysis of Lipid Peroxidation

Lipid peroxidation was quantified using lipid peroxidation kit (Biovision, Milpitas, CA, USA) as per manufacturer’s instructions. Calorimetric measurements were performed at 500 nm using a 96-well-plate spectrometer (Spectra Max 190, Molecular Devices, Sunnyvale, CA, USA) along with 13-hydroperoxyoctadecadienoic acid as a standard. Cellular levels of lipid peroxide were calculated as described by the manufacturer.

### 2.10. Histological and Immunohistochemical Analyses

Liver tissue was fixed in 4% formaldehyde and embedded in paraffin. For histological observation, 4-µm sections were deparaffinized in xylene, rehydrated in an alcohol gradient, and then stained with hematoxylin and eosin (H&E). Immunohistochemical detection of 4-HNE in fixed tissue sections was performed, essentially as previously described [13], using polyclonal antibodies against 4-HNE (Millipore Corporation, Billerica, MA, USA).

### 2.11. Oil Red O Staining

Oil Red O staining was performed as described previously [14]. Briefly, liver sections were fixed with 3.7% formaldehyde at 37 °C for 15 min, after which they were washed twice with phosphate-buffered saline (PBS) and stained with 0.6% (*w*/*v*) Oil Red O solution for 1 h at room temperature. The sections were washed with 60% isopropanol and photographed with a Nikon microscope.

### 2.12. RNA Isolation and Real-Time RT-PCR

Total RNA was isolated from tissues using TriZol (Invitrogen, Carlsbad, CA). cDNA was synthesized from the purified total RNA using a High-Capacity cDNA Reverse Transcription Kit (Applied Biosystems, Foster City, CA, USA). Real-time polymerase chain reaction (RT-PCR) was performed using an SYBR green RT-PCR kit (Applied Biosystems, Foster City, CA, USA) and custom-designed primers (Supplementary Table S1) with qPCR with a sequence detection system (ABI PRISM7900, Applied Biosystems, Foster City, CA, USA).

### 2.13. Statistical Analysis

Mean and standard deviations of the analyzed samples were determined, and groups were compared using one-way ANOVA, followed by *t*-test. A *p* value of <0.05 was considered statistically significant. All observations are expressed as mean ± SEM.

## 3. Results

### 3.1. Analysis of Compounds in CPEs

It is essential to determine the principal components present in the CPEs to assess the CP-associated NAFLD function. The quantification of contents in CPEW and CPEF were analyzed with HPLC which revealed hesperidin, narirutin, and synephrine are the main components of the CPEW and CPEF extracts (Table 1).

**Figure 1:**
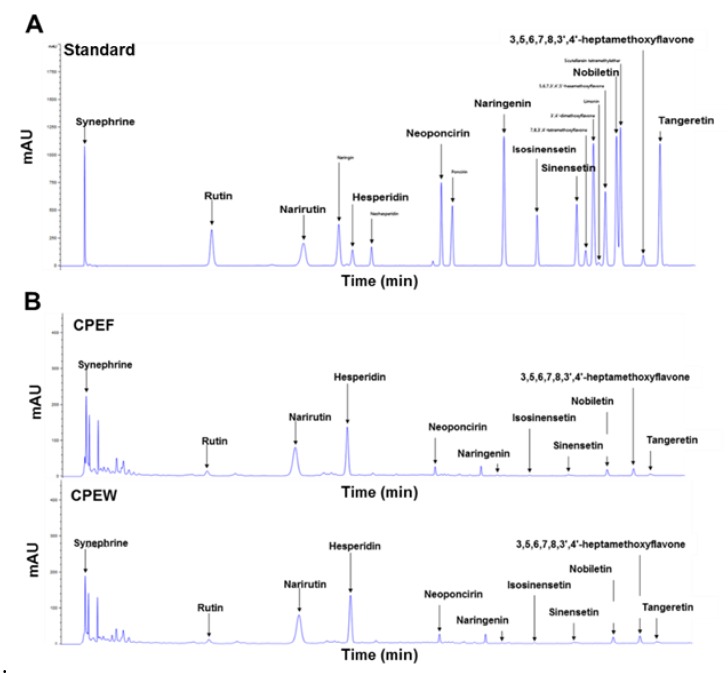
Characteristic analysis of bioactive components in citrus peel extracts by high-performance liquid chromatography at 210 nm. (**A**) Chromatogram showing main components of citrus peel extracts analyzed by standard solution (**B**) Freeze-drying (CPEF) of *citrus peel* water extracts; hot-air-drying (CPEW) of citrus peel water extracts.

Additionally, Chromatogram data on the CPEW and CPEF extracts with the hesperidin, narirutin, and tangeretin standard are also presented in Figure 1A,B. Considering the higher concentration of hesperidin in the extract and its bioavailability in comparison to other components, hesperidin is selected as the effective component for the study.

### 3.2. CPEW and CPEF Prevents HFD-Induced Hepatic Steatosis

**Figure 2:**
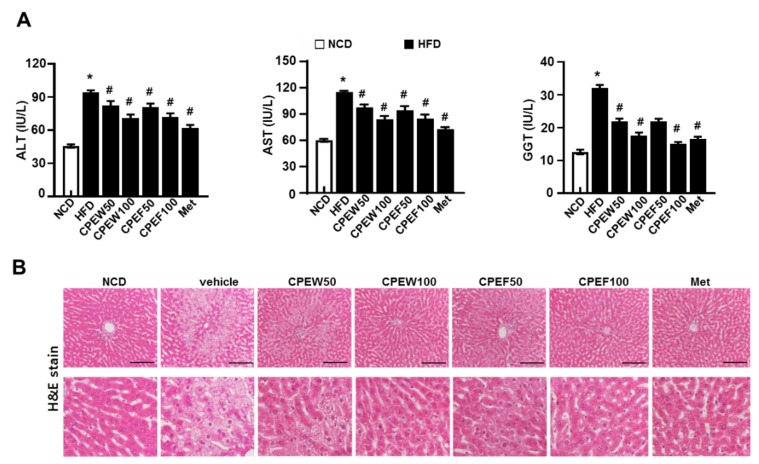
Citrus peel extract protects against HFD-induced hepatic functional damage. Rats were placed on either an NCD or 60% high-fat diet for eight weeks and were supplemented with ultra-pure water, metformin (100 mg/kg), or one of the indicated concentrations of citrus peel (50 and 100 mg/kg). (**A**) AST, ALT, and GGT were measured in HFD-fed rats. (**B**) H&E staining assay was performed using the liver (100 × magnification). Values are mean ± SEM. (*n* = 7~8, * *P* < 0.05 vs. NCD-group, ^#^ *P* < 0.05 vs. HFD-group). CPEW, citrus peel extract hot-air dry; CPEF, citrus peel extract freeze-drying; ALT, alanine aminotransferase; AST, aspartate aminotransferase; GGT, gamma-glutamyl transferase; HFD, high-fat diet; NCD, normal calorie diet.

The in vivo model is suitable to study long-term signal dynamics in a pathophysiologically relevant context. Thus, to investigate the effects of CPEW and CPEF on Hepatic lipotoxicity, rats were fed with HFD to stimulate metabolic disorders for eight weeks. After eight weeks, HFD-fed rats showed significant injuries as demonstrated by increase in serum ALT, AST and GGT (gamma-glutamyl transferase) level than normal-diet group. AST and ALT, hepatotoxicity markers, were significantly downregulated in rats supplemented with CPEW (50 and 100 mg/kg) and CPEF (50 and 100 mg/kg) respectively (Figure 2A). Food intake was also similar in all the groups (Figure S1A). Moreover, to determine the histological effects of CPE on hepatic steatosis, liver sections were stained with H&E to detect lipid accumulation. Histological analysis revealed an extensive increase in histological features of macrovesicular and microvesicular steatosis in HFD-fed rats (Figure 2B).

**Figure 3:**
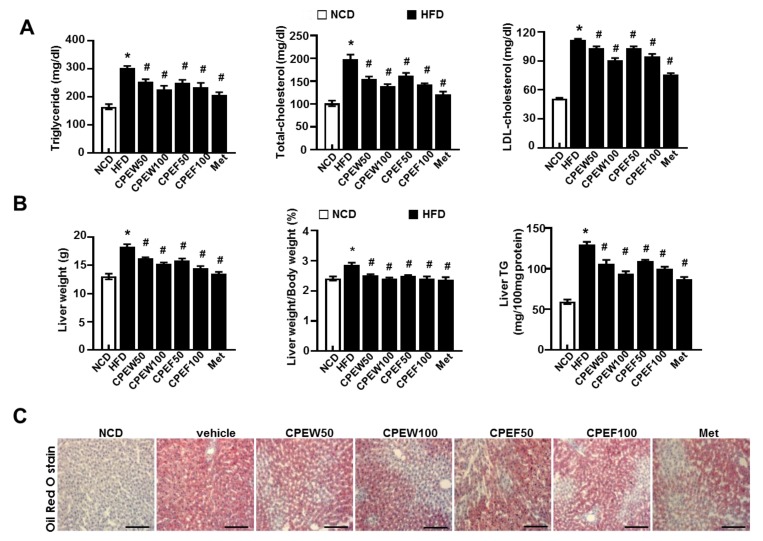
Citrus peel prevents HFD-induced hepatic steatosis. Rats were placed on either NCD or 60% high-fat diet for eight weeks and were supplemented with ultra-pure water, metformin (100 mg/kg), or one of the indicated concentrations of citrus peel (50 and 100 mg/kg). (**A**) Biochemical analysis of plasma samples. Levels of TG, TC and LDL-c were measured in the plasma of rat in different experimental groups. (**B**) Liver weight, and liver weight/body weight and liver TG content were measured 8 weeks after initial CPEW and CPEF administration. (**C**) Liver tissues were subjected to Oil Red O staining. Scale bars, 200 μm. Values are mean ± SEM. (n = 7~8, * *P* < 0.05 vs. NCD-group, ^#^ *P* < 0.05 vs. HFD-group). CPEW, citrus peel extract hot-air dry; CPEF, citrus peel extract freeze-drying; TG, triglycerides; LDL, low-density lipoprotein; HFD, high-fat diet; NCD, normal calorie diet.

Meanwhile, histological stained images showed that supplementation of CPEW (50 and 100 mg/kg) and CPEF (50 and 100 mg/kg) significantly reduced the accumulation of intracellular lipid droplets compared to HFD-fed rats. Furthermore, elevated serum TG, TC, and LDL-cholesterol levels were observed in HFD-fed rats while rats supplemented with CPEW and CPEF showed significant reduction (Figure 3A). Also, liver weight and liver weight/body weight ratio were significantly higher in HFD group than NCD group. In rats supplemented with CPEW (50 and 100 mg/kg) and CPEF (50 and 100 mg/kg), liver weight and liver weight/body weight ratio were similar to NCD group (Figure 3B). CPEW (50 and 100 mg/kg) and CPEF (50 and 100 mg/kg) treatment groups markedly reduced the liver TG levels compared to the HFD-fed group. To further determine the histological effects of CPEW and CPEF on hepatic steatosis, lipid accumulation of liver was detected by the Oil Red O stain of the histological section (Figure 3C). Hepatic intracellular lipid accumulation in HFD-fed rats increased markedly than NCD group, whereas supplementation of CPEW (50 and 100 mg/kg) and CPEF (50 and 100 mg/kg) significantly ameliorated hepatic intracellular lipid accumulation in HFD-fed rats.

### 3.3. CPEW and CPEF Inhibits the mTORC1-ER Stress Response in NAFLD

**Figure 4:**
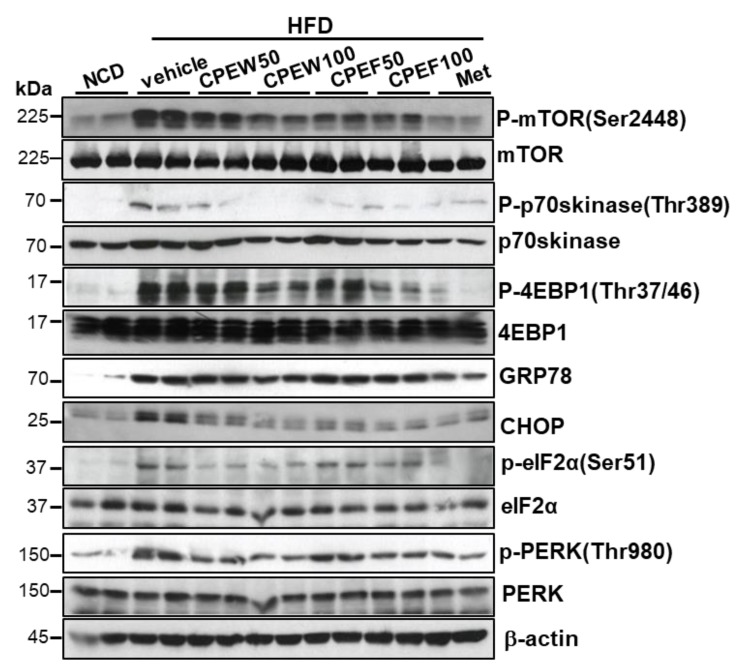
Citrus peel inhibits ER stress and mTORC1 in hyperlipidemic rats. Rats were placed on either an NCD or 60% HFD for eight weeks and were supplemented with ultra-pure water, metformin (100 mg/kg), or one of the indicated concentrations of citrus peel (50 and 100 mg/kg). Immunoblotting using antibodies against p-mTOR, mTOR, p-p70S6K, p70S6K, p-4EBP-1, 4EBP-1, GRP78, CHOP, p-PERK, PERK, p-eIF2α, eIF2α, and β-actin. CPEW, citrus peel extract hot-air dry; CPEF, citrus peel extract freeze-drying; HFD, high-fat diet; NCD, normal calorie diet.

To determine influence of nutrient overload-induced hepatic lipid accumulation in vivo, alteration in ER stress, mTORC1 signaling, and lipid contents in the liver in CPEW and CPEF supplemented HFD-fed rats were examined. Liver tissue of HFD group exhibited activation of mTORC1 signaling as determined by an increase in S6K and 4EBP-1 phosphorylation, which was attenuated by CPEW (50 and 100 mg/kg) and CPEF (50 and 100 mg/kg) treatment (Figure 4). Next, the involvement of ER stress in the CPEW-regulating effect on the dyslipidemia was investigated. The liver tissue of HFD-fed rodents exhibited significant induction of eIF-2α and PERK phosphorylation and downstream target CHOP upregulation. Both of these reactions were attenuated by CPEW (50 and 100 mg/kg) and CPEF (50 and 100 mg/kg) supplementation (Figure 4).

### 3.4. AMPK Activation in CPEW and CPEF Prevents High-Fat Diet-Induced Hepatic Steatosis through Controlling the mTORC1-ER Stress Pathway

**Figure 5:**
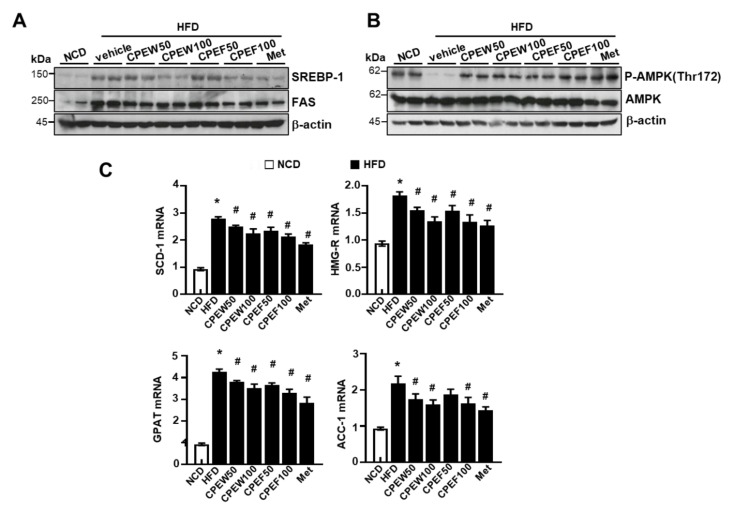
Citrus peel extract inhibits HFD-induced lipid synthesis. Rats were placed on either an NCD or 60% HFD for eight weeks and were supplemented with ultra-pure water, metformin (100 mg/kg), or one of the indicated concentrations of citrus peel (50 and 100 mg/kg). (**A**) Immunoblotting was performed using antibodies against anti-SREBP-1, FAS, (**B**) p-AMPK and AMPK and β-actin. (**C**).

To elucidate the mechanism by which CPEW and CPEF exerted antidyslipidemic effects, protein expressions of de novo lipogenesis-related genes in the liver were examined. The expressions of fatty acid synthase (FAS) and SREBP-1 were higher in the HFD group compared to NCD group, but CPEW (50 and 100 mg/kg) and CPEF (50 and 100 mg/kg) supplementation significantly reduced the expressions of these proteins (Figure 5A). AMPK is suggested to be a therapeutic target for the treatment of obesity due to its role in the regulation of lipid metabolism [8,15]. Therefore, it is necessary to determine whether the activation of AMPK by CPEW and CPEF, prevents HFD-induced intracellular lipid accumulation by controlling the mTORC1-ER stress pathway. HFD-fed-rat group significantly inhibited AMPK phosphorylation at Thr172, indicating the suppression of AMPK activity. Interestingly, the supplementation of CPEW and CPEW restores the AMPK activity (Figure 5B). Similarly, CPEW and CPEF supplementation resulted in the downregulation of mRNA levels of lipogenesis-related genes in liver tissues (Figure 5C).

mRNA expression levels including stearoyl-CoA desaturase-1(SCD-1), 3-hydroxy-3-methylglutaryl CoA reductase (HMG-R), glycerol 3-phosphate acyltransferase (GPAT), and acetyl-coenzyme A carboxylase (ACC) were measured. The quantification analysis of protein expression was performed using the indicated loading control. The quantification analysis of antibody expression was performed using the indicated loading control. CPEW, citrus peel extract hot-air dry; CPEF, citrus peel extract freeze-drying; HFD, high-fat diet; NCD, normal calorie diet.

### 3.5. CPEW and CPEF Suppresses Hepatic Oxidative Stress

**Figure 6:**
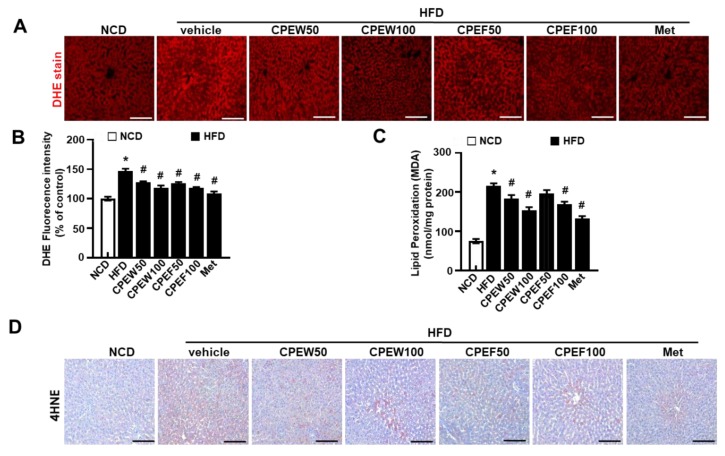
Citrus peel inhibits oxidative stress in hyperlipidemic rats. Rats were placed on either an NCD or 60% HFD for eight weeks and were supplemented with ultra-pure water, metformin (100 mg/kg) or one of the indicated concentrations of citrus peel (50 and 100 mg/kg). In liver tissues, DHE was stained and the fluorescence images were required (**A**) and the fluorescence intensity for superoxide levels was quantified in liver (**B**). In liver lysates, MDA was analyzed (**C**) and in liver section, 4-HNE was stained as described in Materials and Methods (**D**). Values are mean ± SEM. (*n* = 7~8, * *P* < 0.05 vs. NCD-group, ^#^ *P* < 0.05 vs. HFD-group). CPEW, citrus peel extract hot-air dry; CPEF, citrus peel extract freeze-drying; HFD, high-fat diet; NCD, normal calorie diet. Scale bars, 50 μm.

To determine influence of CPEW (50 and 100 mg/kg) and CPEF (50 and 100 mg/kg) on antioxidant capacity, key antioxidant defense components were measured. Supplementation of CPEW (50 and 100 mg/kg) and CPEF (50 and 100 mg/kg) significantly inhibited the superoxide accumulation in HFD group (Figure 6A,B). Also, lipid peroxidation indicated by malondialdehyde (MDA) levels were substantially low in CPEW and CPEF (Figure 6C). Consistently, oxidative marker, 4-hydroxynonenal (4-HNE) was assessed, which was significantly decreased in the presence of CPEW and CPEF (Figure 6D), indicating the controlling role of CPEW and CPEF on the ROS accumulation pattern.

### 3.6. CPEW and CPEF Prevent Palmitate-Induced Lipid Accumulation by Inhibiting the mTORC1-ER Stress Pathway in AML 12 Cells

**Figure 7:**
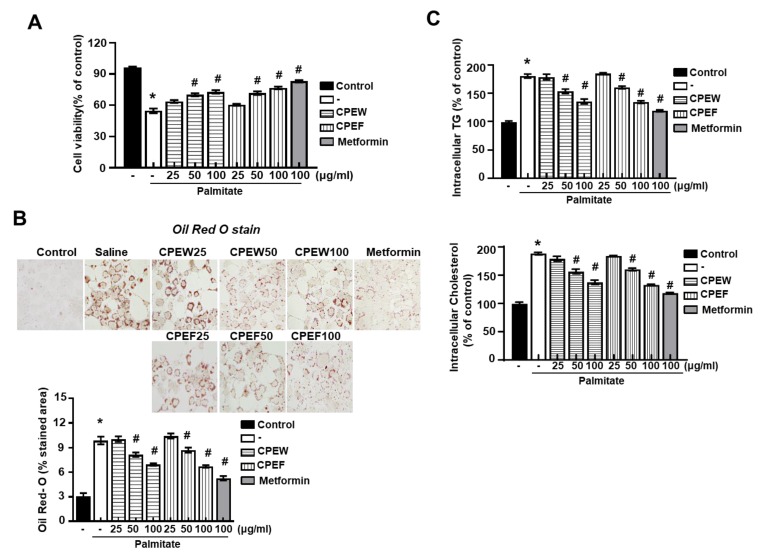
Citrus peel extract regulates hepatic lipid accumulation in palmitate-models. AML12 cells were treated with the indicated concentrations of 50 μg/mL, 100 μg/mL, and 200 μg/mL citrus peel, metformin for 24h for indicated periods, and (**A**) cytotoxicity was determined using the MTT assay. (**B**) Oil-Red-O stain for lipid content staining was measured in the indicated agent treated cells. (**C**) Triglyceride and total cholesterol levels were measured the indicated agent treated cells. Results are mean ± SEM from 3 separate experiments. (^*^ *P* < 0.05 vs. control, ^#^ *P* < 0.05 vs. palmitate). CPEW, citrus peel extract hot-air dry; CPEF, citrus peel extract freeze-drying.

To investigate whether CPEW and CPEF could reproduce the results observed in the HFD-fed rats in vitro, lipogenesis was induced in AML12 cells, *Mus musculus* liver cells. First, the effect of CPEW and CPEF on the cell viability of AML12 cells was determined. To evaluate whether the CPEW and CPEF had a lipid lowering effect on palmitate-treated AML12 cells, the cells were incubated in doses of CPEW (25, 50, and 100 μg/mL) or CPEF (25, 50, and 100 μg/mL). The suppressive effects of CPEW and CPEF on palmitate-induced lipid accumulation in AML12 cells are shown in Figure 7A. Intracellular lipids were then detected using oil red O staining. AML 12 cells exposed to the palmitate showed a clear increase in lipid droplets in the cytosol compared with the BSA control (Figure 7B). Treatment with CPEW (25, 50, and 100 μg/mL) or CPEF (25, 50, and 100 μg/mL) inhibited palmitate-induced lipid accumulation in a concentration-dependent manner. The intracellular TG and total cholesterol content were decreased by both CPEW (25, 50, and 100 μg/mL) and CPEF (25, 50, and 100 μg/mL) in a concentration-dependent manner (Figure 7C).

**Figure 8:**
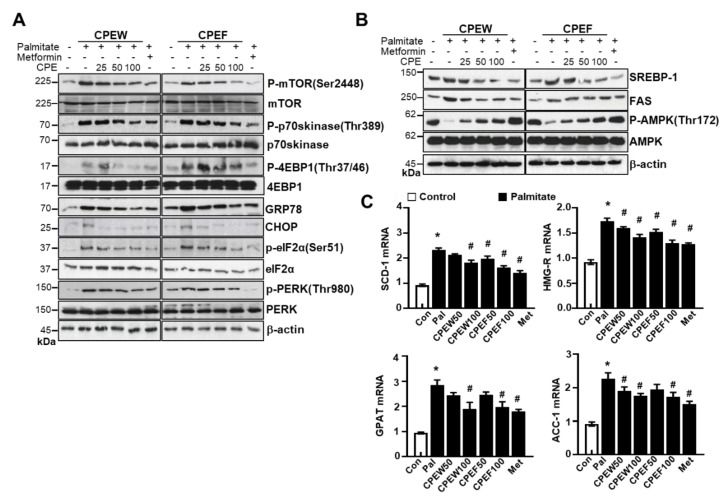
Citrus peel inhibits ER stress in AML12 cells. AML12 cells were treated with the indicated concentrations of 25, 50, and 100 μg/mL citrus peel, metformin for 24 h for indicated periods, and (**A**) immunoblotting was performed using antibodies against p-mTOR, mTOR, p-p70S6K, p70S6K, GRP78, CHOP, p-PERK, PERK, p-eIF2α, eIF2α, and β-actin. (**B**) Immunoblotting was performed using antibodies against SREBP-1, FAS, p-AMPK, AMPK and β-actin. (**C**) mRNA expression levels including stearoyl-CoA desaturase-1 (SCD-1), 3-hydroxy-3-methylglutaryl CoA reductase (HMG-R), glycerol 3-phosphate acyltransferase (GPAT), and acetyl-coenzyme A carboxylase (ACC) were measured as described in Materials and Methods. CPEW, citrus peel extract hot-air dry; CPEF, citrus peel extract freeze-drying.

To determine the mechanisms underlying nutrient overload-induced hepatic lipid accumulation in vitro, we analyzed alteration of mTORC1 signaling, ER stress, and lipid contents in CPEW and CPEF-treated and palmitate-induced AML12 cells. p-4EBP-1, p-mTOR, and p-p70s kinase were significantly increased upon Palmitate treatment. In contrast, treatment with CPEW and CPEF significantly reduced the phosphorylation of these proteins in palmitate treatment. Palmitate treatment induced ER stress, indicated by CHOP, GRP78, PERK, and eIF2a in AML12 cells whereas co-treatment with CPEW and CPEF significantly inhibited PERK and eIF2α phosphorylation, suppressing expression of GRP78 and CHOP upon palmitate treatment (Figure 8A). Next, influence of CPEW and CPEF treatment on AMPK activity in AML12 cells was assessed. Palmitate treatment exhibited a reduction in AMPK phosphorylation than control, suggesting inhibition of AMPK. The AMPK activity was restored by CPEW and CPEF (Figure 8B). Notably, palmitate-induced nuclear SREBP-1 and FAS levels were abrogated by CPEW and CPEF, indicating the role of CPEW and CPEF on the hepatic lipid synthesis signaling. In comparison with the control, palmitate-treatment increased mRNA expression level of genes related to fatty acid synthesis, such as SCD-1, HMG-R, GPAT and ACC, respectively (Figure 8C). Supplementation with CPEW, CPEF, and metformin reversed the elevations of mRNA factors involved in fatty acid synthesis.

### 3.7. The Major Components of CPE and Hesperdin Regulate AMPK Activation in AML12 Cells.

**Figure 9:**
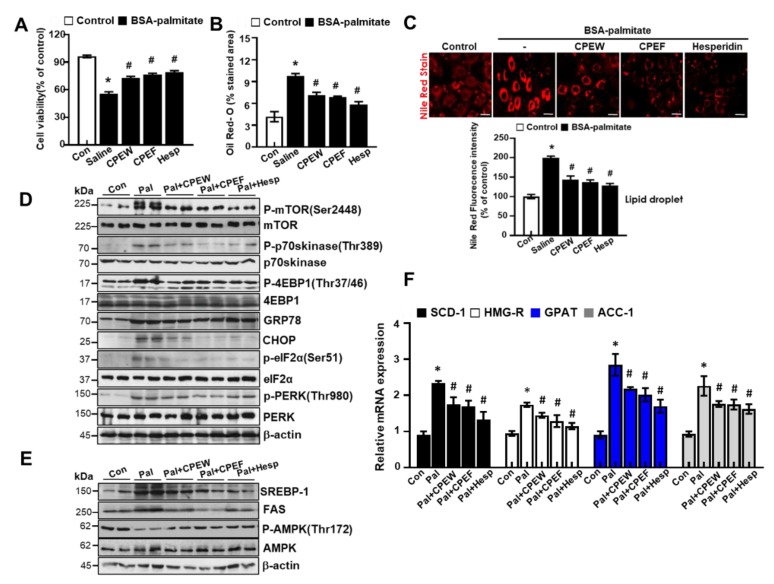
The major components of citrus peel regulate palmitate-induced steatosis in AML cells. (**A**) AML12 cells were treated with 100 μg/mL CPEW, 100μg/mL CPEF or 50 μM hesperidin or 24 h for the indicated periods, and cytotoxicity was determined using the MTT assay. (**B**) Oil-Red-O stain for lipid content staining was measured in palmitate-treated cells. (**C**) Nile Red stain for lipid content staining was measured in palmitate-treated cells. AML12 cells were treated with 100 μg/mL CPEW, 100 μg/mL CPEF, or 50 μM hesperidin for indicated periods, and the expression of proteins of interest was determined by western blotting. (**D**) Immunoblotting was performed using antibodies against p-mTOR, mTOR, p-p70S6K, p70S6K, GRP78, CHOP, p-PERK, PERK, p-eIF2α, eIF2α, and β-actin. (**E**) Immunoblotting was performed using antibodies against SREBP-1, FAS, p-AMPK, AMPK and β-actin. (**F**) mRNA expression levels including stearoyl-CoA desaturase-1 (SCD-1), 3-hydroxy-3-methylglutaryl CoA reductase (HMG-R), glycerol 3-phosphate acyltransferase (GPAT), and acetyl-coenzyme A carboxylase (ACC) was measured. Results are mean ± SEM from 3 separate experiments. (^*^P < 0.05 vs. control, ^#^*P* < 0.05 vs. palmitate). CPEW, citrus peel extract hot-air dry; CPEF, citrus peel extract freeze-drying.*.

We tested the effects of hesperidin, the major active components of CPE [16], on palmitate-induced lipotoxicity in AML12 cells. First, CPEW, CPEF and hesperidin showed a protective effect against palmitate-induced cell death (Figure 9A) and decrease in lipid droplets in cytosol was observed than control (Figure 9B,C). mTORC activation was examined in the presence of CPEW and CPEF and hesperidin. Palmitate significantly reduced the phosphorylation of mTOR, p-p70s6kinase, and 4EBP-1, whereas CPWH and CPEF and hesperidin reduced mTORC activation in AML12 cells. Also, co-treatment with hesperidin significantly blocked the ER stress signals (Figure 9D). To elucidate the mechanism by which CPEW, CPEF and hesperidin-exerted antidyslipidemic effects, lipogenesis-related gene levels were analyzed in AML12 cells. The expressions of SREBP-1 and FAS were enhanced in the palmitate treatment than the control, but those were significantly decreased under the CPEW, CPEF or hesperidin. The CPEW, CPEF and hesperidin treatment also increased p-AMPK/AMPK (Figure 9E), a main controlling upstream signaling on mTORC1 and ER stress and its associated hepatic lipogenesis genes [17]. Supplementation with CPEW, CPEF, and hesperidin reversed the elevations of mRNA factors involved in fatty acid synthesis. (Figure 9F).

## 4. Discussion

Considering the potential therapeutic values of CPEs, it was hypothesized that supplementation of CPEs might have positive influence on regulating high-fat diet-induced NAFLD and hepatic steatosis. Thus, in the study, efforts were made to evaluate the effects of CPEW and CPEF supplementation on high-fat diet-induced NAFLD and hepatic steatosis. Quantification of CPEs revealed narirutin and hesperidin as major components of CPEW and CPEF. However, previous studies reported that hesperidin bioavailability is higher than the narirutin [18]. Thus, considering the concentration of hesperidin in CPEs and its bioavailability it can be considered as a key component in the investigation. Upon investigation, it was observed that CPEW and CPEF supplementation significantly reduced serum total cholesterol, TG level, and liver TG content and also suggested that AMPK activation is the main factor behind the protective effect of CPEW and CPEF in high-fat diet-induced NAFLD, which otherwise would be due to mTORC1 activation and subsequent protein folding load involved in ER stress response. CPEW and CPEF reduced hepatic weight gain through inhibition of lipid accumulation in the liver tissue without reducing body weight (Supplementary Figure S1B). Consistently, CPEW and CPEF supplementation reduced both hepatocyte steatosis and fat accumulation (Figure 2 and Figure 3). These observations can be attributed to the presence of Hesperidin in the CPEs. Recently, a clinical investigation shown that hesperidin supplementation reduced hepatic enzymes and liver steatosis [19] correlating with present investigational observation. However, a mechanism of action needs to be realized for the development of therapeutic strategies. It is a well-known fact that a high level of free fatty acids leads to constitutive activation of mTOR signaling, a pathway related to the advancement of conditions such as NAFLD and obesity [20,21]. ER integrity and cause of UPR is challenged by conditions where mTORC1 signaling pathway has input from several upstream pathways, including nutrient-sensing pathways with cell growth and metabolism [13]. Consistent with previous findings that the knockout of TSC1 or TSC2 induces UPR, resulting in mTOR-associated feedback inhibition of insulin action [22], it is noted that nutrient overload induces ER stress response in an mTORC1-dependent manner. In addition, chronic liver ER stress was observed in the steatosis of mice strains with genetically deficient in leptin as well as genetically modified IRE1/XBP1, PERK/eIF2α and ATF6 signaling pathways [17,20]. The ER stress response is one of the main features of pathological conditions associated with obesity and NAFLD [23,24,25]. However, the mechanism by which CPEW and CPEF supplementation inhibit the ER stress response in NAFLD etiology is not clear. High levels of free fatty acids are known to cause constitutive mTOR signal activation, a process associated with diseases such as diabetes and obesity [21,26]. Indeed, when treating palmitate-incubated hepatocyte with CPEW and CPEF, there was significant decrease in mTORC1/ER stress axis (Figure 4). On HFD, increased PERK/eIF2α branch is associated with the upregulation of SREBP-1 and its target protein FAS as well as with lipid accumulation (Figure 5). The activation of AMPK, well-known physiological inhibitor of the energy-consuming mTOR signaling pathway [13,14,15], is a possible mechanism to explain the inhibition of mTOR/ER stress axis in NAFLD. Inactivation of AMPK activates the mTORC1 signaling pathway in HFD diet rats, leading to lipid accumulation. Supplementation of CPEW and CPEF restored AMPK activity, inhibited mTORC1 signaling, and reduced lipid metabolism (Figure 4). The activation of AMPK by CPEW and CPEF reduced p-mTOR, p-S6K and p-4-EBP-1 levels, attenuated ER stress, and inhibited hepatic dyslipidemia *in vivo*. CPEW and CPEF reduced hepatic lipid accumulation caused under excess nutrient conditions, indicating that the PERK/eIF2α branch plays a key role in lipid metabolism via the regulation of SREBP-1 and FAS. These observations suggest that the PERK/eIF2α branch plays a crucial role in the development of dyslipidemia. CPEW and CPEF were shown to influence mTORC1 status, thus controlling the ER stress response and redox imbalance indicating the involvement of mTORC1 activation and protein folding in pathological mechanisms of hepatic dyslipidemia. Another important finding of the study is that CPEW and CPEF supplementation alleviates hepatic steatosis by modulating ROS accumulation associated with ER redox disorder and liver dysmetabolism associated with ER stress. The main factor in the early development of obesity and metabolic diseases is known as oxidative stress, which improves metabolic and vascular effects of NAFLD [27]. Citrus plants are rich in dietary flavonoids and are used for their pharmacological effects in food and medicine metabolic syndrome and cardiovascular and neurodegenerative diseases [16,28]. Results showed increased oxidative and ER stresses in HFD-fed rats, as evidenced by increased levels of hepatic 4-HNE, serum MDA levels and UPR, i.e., induction of PERK/eIF2 branch. The CPEW and CPEF also prevents oxidative stress caused by upregulation of the activity of antioxidant enzymes such as MDA (Figure 6). The results suggested that CPEW and CPEF control ER stress by alleviating the ER-oxido-reduction folding disturbance, subsequently diminishing the ER stress-associated ROS levels. Further, CPEW and CPEF inhibit lipid metabolism imbalance by alleviating hepatic steatosis through oxidative stress relief, regardless of whether or not the CPEW and CPEF is stressed (Figure 8). Citrus are rich in flavonoids, majorly contributed by flavanones such as hesperidin, hesperitin, naringin and naringenin; polymethoxylated flavones such as nobiletin and tangeretin [1]. Citrus flavonoids have antioxidant, anti-inflammatory, and antitumor activities [2,16]. Hesperidin, a major compound of citrus peel [29], suppresses cell death through regulation of AMPK activation [30]. Along with other components in citrus peel, mTOR was inhibited by hesperidin. Furthermore, in vitro data indicate that hesperidin reduced the expression of the ER stress-related proteins in PERK, eIF2α, GRP78, and CHOP, and mTOR/AMPK axis recovery (Figure 9). Thus, the active component of citrus peel, hesperidin, has similar effects on the regulation of mTOR/AMPK axis, related unfolded-protein loading, ER stress, and cell death. In this study use of freeze dried and air dried extractions methods might have made unnecessary complications in data presentation. However, this study was designed to differentiate the potential impact of extraction method on the availability of bioactive compounds and their influence on lipid steatosis. Fortunately, both methods were efficient and yielded almost similar observations.

## 5. Conclusions

In conclusion, the study demonstrates that supplementation of CPEW and CPEF efficiently inhibits fatty liver development and hepatotoxicity in high-fat diet-induced NAFLD, and also prevents abnormal lipid accumulation in vivo by regulating AMPK activation and the alleviation of mTORC1-ER stress. These experimental evidences shed light on underlying protective mechanism, thus paving the way for developing novel strategies to prevent complications of NAFLD.

## Figures and Tables

**Table 1:** Components of the Citrus peel extract.

Compounds	CPEF (mg/100g)	CPEW (mg/100g)
Synephrine	456	373
Rutin	103	87
Narirutin	1028	1034
Naringin	ND	ND
Hesperidin	734	898
Neohesperidin	ND	ND
Neoponcirin	79	81
Poncirin	ND	ND
Naringenin	5	3
Isosinensetin	5	5
Sinensetin	4	4
7,8,3′,4′-tetramethoxyflavone	ND	ND
3′,4′-dimethoxyflavone	ND	ND
Limonin	ND	ND
5,6,7,3′,4′,5′-hexamethoxyflavone	ND	ND
Nobiletin	45	46
Scutellarein tetramethylether	ND	ND
3,5,6,7,8,3′,4′-heptamethoxyflavone	69	70
Tangeretin	5	6

CPEW: Citrus peel extract through hot-air drying; CPEF: Citrus peel extract through freeze-drying.

## Supplementary Materials

The following are available online at https://www.mdpi.com/2072-6643/12/3/673/s1, Figure S1: The effects of Citrus peel extract on body weight and food intake in high fat diet-induced hepatic steatosis in rat. Table S1: Sequences for Primers Used in Real-time RT-PCR.
