# Supplementary material for: Citrus Peel Extract Ameliorates High-Fat Diet-Induced NAFLD via Activation of AMPK Signaling
Source: Nutrients. 2020 Mar 1;12(3):673. doi: 10.3390/nu12030673 (PMC7146518; doi:10.3390/nu12030673)
Supplement: Supplementary file 1 [file nutrients-12-00673-s001.pdf]

Supplementary information

Table S1. Sequences for Primers Used in Real-time RT-PCR.

Genes	Rat	Mouse
**SCD-1**	Foward-5'-atcccctcctccaaggtcta-3'	Forward-5'-gaggcctgtacgggatcata-3'
	Reverse-5'-cgggcccattcatatacatc-3'	Forward-5'-tgagagaagaagaagccacgg-3'
**HMG-R**	Forward-5'-agaatatagcgcgtgggatg-3'	Forward-5'-aatgccttgtgattggagttg-3'
	Reverse-5'-gacatacagccaaagcagca-3'	Forward-5'-cagacccaaggaaaccttagc-3'
**GPAT**	Forward-5'-cagcgtgattgctacctgaa-3'	Forward-5'-caacaccatccccgacatc-3'
	Reverse-5'-ctctccgtcctggtgagaag-3'	Reverse-5'-ccgcagcattctgataacgc-3'
**ACC**	Forward-5'-gtttggcctttcacatgaggtcc-3'	Forward-5'-acagtggagctagaattggac -3'
	Reverse-5'-gtggggatacctgcagtttga-3'	Reverse-5'-actttccgaccaaggactttg-3'
**GAPDH**	Forward-5'-cctggagaaacctgccaagtat-3'	Forward-5'-aatggtgaaggtcggtgtg -3'
	Reverse-5'-ctcggccgcctgctt-3'	Reverse-5'-gtggagtcatactggaacatgtag-3'

SCD: Stearoyl-CoA desaturase -1 ,HMG-R: 3-hydroxy-3-methylglutaryl CoA reductase, GPAT: Glycerol 3-phosphate acyltransferase ,ACC: acetyl-coenzyme A carboxylase .

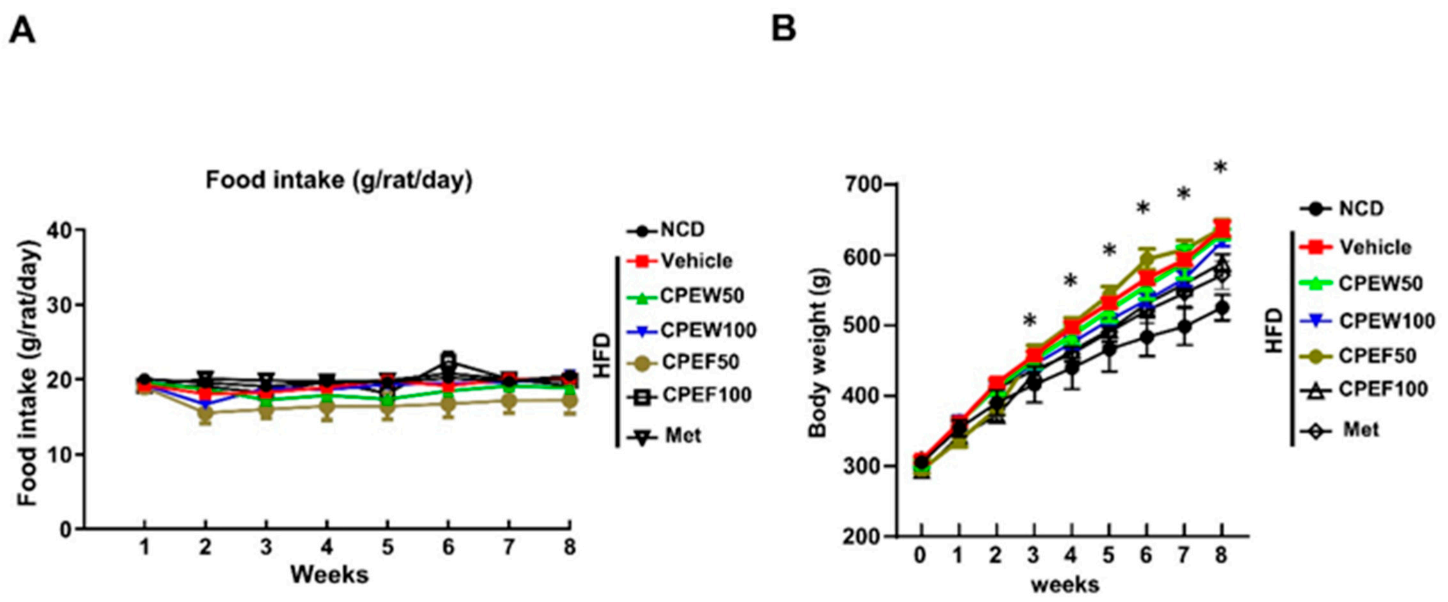

Figure S1. The effects of Citrus peel extract on body weight and food intake in high fat diet-induced hepatic steatosis in rat.
